# LF Power of HRV Could Be the Piezo2 Activity Level in Baroreceptors with Some Piezo1 Residual Activity Contribution

**DOI:** 10.3390/ijms24087038

**Published:** 2023-04-11

**Authors:** Balázs Sonkodi

**Affiliations:** Department of Health Sciences and Sport Medicine, Hungarian University of Sports Science, 1123 Budapest, Hungary; bsonkodi@gmail.com

**Keywords:** delayed-onset muscle soreness, heart rate variability, low-frequency power, baroreflex, Piezo2 ion channel

## Abstract

Heart rate variability is a useful measure for monitoring the autonomic nervous system. Heart rate variability measurements have gained significant demand not only in science, but also in the public due to the fairly low price and wide accessibility of the Internet of things. The scientific debate about one of the measures of heart rate variability, i.e., what low-frequency power is reflecting, has been ongoing for decades. Some schools reason that it represents the sympathetic loading, while an even more compelling reasoning is that it measures how the baroreflex modulates the cardiac autonomic outflow. However, the current opinion manuscript proposes that the discovery of the more precise molecular characteristics of baroreceptors, i.e., that the Piezo2 ion channel containing vagal afferents could invoke the baroreflex, may possibly resolve this debate. It is long known that medium- to high-intensity exercise diminishes low-frequency power to almost undetectable values. Moreover, it is also demonstrated that the stretch- and force-gated Piezo2 ion channels are inactivated in a prolonged hyperexcited state in order to prevent pathological hyperexcitation. Accordingly, the current author suggests that the almost undetectable value of low-frequency power at medium- to high-intensity exercise reflects the inactivation of Piezo2 from vagal afferents in the baroreceptors with some Piezo1 residual activity contribution. Consequently, this opinion paper highlights how low-frequency power of the heart rate variability could represent the activity level of Piezo2 in baroreceptors.

## 1. Introduction

Power spectral analysis of heart rate variability (HRV) measurements is a widely accepted method for analyzing cardiac autonomic function. There has been a long-lasting scientific debate on whether the low-frequency (LF) power of the HRV reflects the sympathetic loading, whereas Goldstein et al. elegantly reasoned that it is rather a measure of how the baroreflex modulates the cardiac autonomic outflows [1]. It is known that cardiac parasympathetic activity measured by HRV returns to pre-exercise levels only 1 to 2 days after strenuous exercise [2] with a time overlap of the suspected acute proprioceptive terminal Piezo2 channelopathy [3,4]. Furthermore, Perini and Veicsteinas showed in their study 20 years ago that LF power is dependent on exercise intensity with no change at low intensity, but the LF power diminishing to almost imperceptible values at medium to high intensity [5].

The association between LF rhythm and arterial pressure variance through the baroreceptor was first reported by Saul [6]. It was a recent major scientific breakthrough when the Piezo ion channels were discovered by a team of the Nobel Prize laureate Ardem Patapoutian [7], not to mention when the Piezo1 and Piezo2 channels were identified in the baroreceptors by their team with a principal role of detecting blood pressure (BP) and contributing to the baroreceptor baroreflex [8]. Resetting the baroreflex during muscular work in order to adjust arterial pressure control through the chemoreflex and mechanoreflex has long been hypothesized [9]. Notably, both mechano and indirect chemo transducing characteristics of somatosensory Piezo2 in exercise were recently emphasized [4]. Moreover, reflex changes to heart rate due to exercise are essentially influenced by muscle afferent activity through mechano- and metaboreceptors [10].

The exact molecular basis of this mechanotransduction in baroreceptors had been unknown for more than 80 years [11]. Piezo ion channels are stretch- and force-gated excitatory cationic channels with a principal role in proprioception [12], and it was recently demonstrated in mice that were not able to compensate for small changes in BP in the absence of neuronal Piezo1 and Piezo2 [8]. Even more importantly, the baroreflex could be induced by optogenetic activation of the Piezo2 sensory afferents [8].

Piezo2 channels in sensory terminals are abruptly activated and go through inactivation when these terminals go from excitation to hyperexcitation [13], in order to prevent pathological hyperexcitation. This opinion manuscript theorizes why this characteristic feature of Piezo2 could be telling about its principal role in the LF power representation of HRV; hence, the Piezo2 activity level could be the long-searched measure of LF power of HRV with some Piezo1 residual activity contribution.

## 2. Piezo Ion Channels and Baroreceptors

The nonselective Piezo ion channel proteins are the largest transmembrane proteins with force-gated excitatory mechanosensitive properties, featuring numerous transmembrane segments and pore-forming subunits [7,14]. They are responsible for converting mechanical stimuli into biochemical life-sustaining signals, such as touch sensation, proprioception, and cardiovascular regulation [15]. These mechanotransducer Piezo proteins are mechanically activated, especially by stretch and shear stress [16,17]. They have two types in human, Piezo1 and Piezo2, with similar interesting properties, e.g., their propeller blades [18]. Nonetheless, their exact physical and functional properties are far from entirely known, such as pore formation, mechanical force detection, and gating [14].

On the periphery, Piezo2 ion channels participate in homeostasis maintenance in somatosensory neurons [19,20], in contrast to Piezo1 ion channels that contribute to homeostasis in neuromodulator peripheral cells, e.g., in BP regulation [8] and regulation of osmolarity [21]. In addition, Piezo1 channels also play a role in cell alignment due to their shear stress sensor capability [22,23]. Hence, these Piezo1-containing peripheral tissues cells react like cellular mechanoreceptors, and they can act as neuromodulators through the crosstalk with somatosensory terminal Piezo2 ion channels [24].

Animal research has shown that impairing baroreceptors elevates BP with increasing variability; however, even more importantly, these animals could not maintain fine reflex adjustments due to BP changes instigated by orthostatic change or physical activity [11]. Part of the aortic arch is densely innervated by vagal afferents, and a portion of these contain Piezo2 channels [25,26]. Even more importantly, it was shown through optogenetic manipulation that indeed only Piezo2-containing vagal afferents are responsible for BP detection in the baroreceptors by invoking the baroreflex [26]. The aortic arch enwrapping claw-like innervation of these Piezo2-containing vagal afferents [26] behaves very much like lanceolate endings in the skin responsible for gentle touch sensation [27], as highlighted by Ghitani and Chesler [25]. Therefore, these Piezo2-containing vagal afferents innervating baroreceptors can sense the stretch of the aortic wall precisely when the BP changes, thereby instigating fine reflex adjustments in BP.

Research is emerging about the pressure pulse-detecting capability of Piezo2 via baroreceptors and its abrupt mechanotransmission, unlike neurotransmitter-based detection, throughout the supraspinal level [28]. This supraspinal detection and mechanotransmission may be analogous to that reported by Zeng et al. on the periphery, where Piezo2 channels of the baroreceptors detect BP and contribute to the baroreceptor baroreflex on the spinal level [8]. An interesting theory was recently published, suggesting that Piezo2 proteins generate intrinsic resonance in supraspinal neurons in which they are located, in order to entrain with breathing and cardiac cycles [28]. This proposed entrainment mechanism could exist on the periphery, as well as spinally, constructing the supraspinal and spinal synchronizations of these cycles. This theoretical synchronization could be extremely fast according to Pascal’s law, reaching almost the speed of sound [28,29]. Noteworthily, the Piezo2 presentation supraspinally is limited to certain neuron types and locations [28], e.g., hippocampal excitatory pyramidal cells [28], which are capable of processing spatial, contextual, and emotional cues based on motor and sensory inputs, not to mention the role of the hippocampus in new memory consolidation [30]. Indeed, certain pyramid cells represent the pacemaker region for synchronization in the hippocampus [31]. However, a detailed discussion of the aforementioned mechanisms with Piezo2 contribution is not the main objective of this paper.

The current author suggests that Piezo2-containing vagal afferents from the baroreceptors could be in crosstalk with the vagal afferents of the lung and heart. As a result, high activation of Piezo2 (low excitation) on the vagal afferents of the baroreceptors could mean high synchronization, whereas the quality of synchronization would diminish at a low Piezo2 activity level (high excitation). Once Piezo2 is inactivated and the sympathetic load still increases, then the Piezo2-containing vagal afferents of the baroreceptors and lungs are suggested to be desynchronized. Consequently, deep and slow breathing stimulates the Piezo2-containing vagal afferents and promotes the aforementioned entrainment, while rapid and shallow breathing could lead to a point where the LF peak might not even form, indicating the aforementioned disentrainment. Moreover, this vagal afferent desynchronization could also leave the cardiac sympathetic activation unopposed. The critical ion channels of the heart rhythm controlling pacemaker cells in cardiac sympathetic activation could be the Cav1.3 channels [32], and their pacing could be unopposed in the aforementioned vagal afferent desynchronization, as first described by Cohen and Taylor [33]. However, the picture is not straightforward, since it is known that, even after cardiac deafferentation in transplant patients, approximately 2–8% of normal R–R interval variability still remains within the respiratory cycle [34]. One explanation could be that Piezo1 activation from peripheral cells may exert a residual effect in the Piezo2 inactivated state, considering the existence of the theoretical functional interaction between Piezo1 and Piezo2 [24,35]. Noteworthily, this Piezo1–Piezo2 crosstalk could cease to exist only in the presence of a Piezo channelopathy, as previously postulated [24]. Therefore, mechanostransduction from the Piezo1 channels of cardiomyocytes due to the stretch of the atria by cardiac filling and the changes in thoracic pressure as a consequence of respiration could still exert the aforementioned residual effect, with Bainbridge suggesting as early as 1930 that mechanical load has an autonomic influence on the link between respiration and pulse rate [36].

## 3. LF Power of HRV

**Figure 1:**
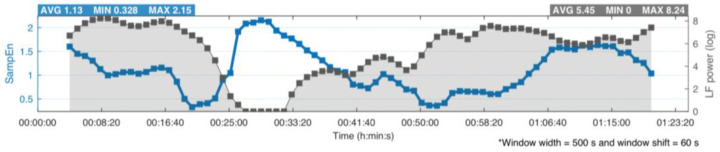
LF power (log) of HRV presented with a black dotted line. Medium- to high-intensity exercise decreases the LF power of HRV to imperceptible values [5], exhibited at around 26 min. The figure presentation is only demonstrative, constructed using Kubios HRV software (Kubios Oy, Kuopio, Finland, https://www.kubios.com, accessed on 23 February 2023).

There are two major rhythmic components of HRV, and these can be measured by power spectral analysis. The high-frequency (HF) domain at 0.19 Hz is commonly considered to represent cardiac vagal activity associated with respiratory activity, in contrast to the LF component at 0.09 Hz that is controversially viewed to represent sympathetic activity [5]. However, it has been long shown that neither HF nor LF power entirely embodies the vagal activity decrease or the sympathetic activity increase during exercise [5]. Correspondingly, at medium- to high-intensity exercise, when the LF power decreases to almost 0 (Figure 1), the sympathetic activity still increases. The current author suggests that this divergence is the point at which the excitatory Piezo2 channels in the baroreceptor are inactivated.

Indeed, sympathetic loading diminishes the feedback control of muscle length by muscle spindles [37,38,39]. These muscle spindles contain primary proprioceptive sensory afferents with Piezo2 in their terminals [40,41], and these terminal Piezo2 channels are proposed to contribute principally to the static phase firing sensory encoding of the stretch reflex [42]. Moreover, it is emphasized that, during an acute stress response (ASR), the fine motor movements are traded for the flight-and-fight response [43]. However, this functional proprioceptive feedback loss could come earlier than an ASR inducement, i.e., when the hyperexcited Piezo2 channels are inactivated. At this stage ASR is not yet initiated, and voltage-gated sodium ion channels, such as Nav1.1 in particular, could take over the propagation of proprioceptive input with lessened encoding quality. Indeed, when Piezo2 is inactivated, e.g., due to prolonged muscle stretch, then the Nav1.1 channel communicates the rhythmic firing [44]. Correspondingly, it was shown that LF power decreases even at lower intensities due to prolonged constant load task, in contrast to incremental ones [5].

Furthermore, muscular feedback, position sense, orthostasis, and proprioception truly do have relevance in the LF power rhythm. For example, the height of LF power is steeper and increased in sitting compared to supine, and a 20–50% increase could be observed in the LF peak when the subject took a sitting position after a supine one [5]. The current author interprets these results as sitting requiring more proprioception and, hence, greater position sense and higher Piezo2 activation, in contrast to supine, when position sense and Piezo2 activation are negligible. Even more interestingly, the LF power diminished at high altitude compared to sea level in the sitting position, while LF power showed a marked increase at high altitude compared to sea level in the supine position [5]. Noteworthily, under eccentric contractions, the muscle spindle feedback was loaded in order to support the body against gravity [45,46]. This telling antigravitational fine-tuning feature of muscle spindles through the stretch reflex, which involves Piezo2 at the proprioceptive terminals, could explain the aforementioned phenomena at high altitude. Accordingly, the decrease in gravity and resultant lessened activation of Piezo2 could be the reason why the LF power was lower at high altitude. In contrast, the diminished gravity could activate the mostly inactivated Piezo2 and elevate LF power from almost 0 in a supine position due to the differential compared to the set baseline gravity homeostatic threshold. Moreover, Perini et al. observed no difference in LF values in the same position in air or head-out immersion [47], and this could be explained by the loss of gravity and resultant decreased Piezo2 activation.

Another compelling finding is that LF power was lower in ergometer cyclists compared to sedentary subjects [5]. Certainly, this difference could not be explained by a lower level of sympathetic activity, because the ergometer cyclist had a higher LF power prior to exercise [5]. Nevertheless, ergometer cycling and concentric contraction-based training could take the load off muscle spindles, as well as alleviate the position sense and proprioceptive loading, thus relieving the activation of Piezo2 in the proprioceptive terminals [48,49]. This analogy could also hold in the case of steady state exercise, by diminishing the contribution of eccentric contractions and, hence, muscle spindle involvement, due to the sustainment of the same range of exercise intensity. Hence, the lower LF power measure could be explained for ergometer cyclists [5] and in steady-state exercise [50] through the contribution of decreased Piezo2 activation at intrafusal proprioceptive terminals, indicating less entrainment loading. Indeed, non-neural factors, likely humoral, could play a role in the maintenance of heart rate during steady-state exercise [50]. In line with the above theory, Perini and Veicsteinas long suspected the involvement of muscle afferents [5]. Furthermore, higher LF power prior to ergometer cycling [5] and in post-exercise recovery after steady-state exercise [50] could represent the higher synchronizing capacity of Piezo2-containing vagal afferents in the baroreceptors and, hence, higher Piezo2 activity level.

## 4. Proprioceptive vs. Piezo System

Remarkably, the proprioceptive system is neuro-energetically proposed to have resource limitations [42]. Therefore, if it is overloaded neuro-energetically at one spinal segment, then it will detract energy from other segments. The most neuro-energy-consuming segment will be affected most in this neuro-energy reallocation, i.e., the head/neck area [48]. This resource limitation has relevance in exercise at high intensities, as well as in neurodegeneration or aging [46,48]. Furthermore, it has special relevance when Piezo2, the principle proprioceptive channel [12], is micro-injured, because it is suggested to enhance this neuro-energetic reallocation process in order to increase postural stability in the form of compensatory exaggerated contractions on the affected segment [48].

It was addressed recently that the crosstalk between Piezo2 and Piezo1 channels constructs a Piezo system [24], which is not a perfect overlap with the proprioceptive system. For example, ion channels other than Piezo ion channels contribute to proprioception. However, Piezo1 is able to sense not only in a spatially restricted fashion, but on a whole-body level to increase performance (e.g., exercise), in parallel with the maintenance of cardiovascular homeostasis [51,52]. Moreover, it is known that intensive and prolonged exercise causes a plasma shift and hemoconcentration, and the contribution of the Piezo system to this mechanism was suggested [52]. The current author proposes that, when the intensity of exercise reaches a medium to high level, then Piezo2, with the excitatory contribution of Piezo1 through their crosstalk, in the baroreceptors will become hyperexcited due to the plasma shift and hemoconcentration; over time, Piezo2 becomes inactivated in order to prevent pathological hyperexcitation. Noteworthily, Zeng et al. showed in animal research the coordinated contribution of Piezo1 and Piezo2 to the baroreflex [8]. In summary, Piezo2 inactivation is postulated to represent the aforementioned decrease in LF power of HRV to an imperceptible level. The residual insignificant value could be the aforementioned mechanical input from Piezo1.

It was recently emphasized that the essential role and heavier loading of proprioception (or, more importantly, the Piezo system) gained special ontogenetic relevance during erection to an upright posture when bipedality evolved in humans [53]. In a strange syndrome, an erection-related micro-injury of Piezo2 is proposed as the primary proprioceptive damage, and a consequence of its acute symptoms is unsurprisingly transient autonomic imbalance [3]. Irreversible proprioceptive terminal Piezo2 microinjury is also suggested in a lethal motoneuron disease called amyotrophic lateral sclerosis (ALS) [54,55], and the autonomic impairment in this condition has long been reported [56]. Furthermore, this accelerated lethality in ALS is associated with terminal circulatory and respiratory collapse and might not be due only to muscular insufficiency. Indeed, the aforementioned proposed involvement of Cav1.3 channels in the absence of activated Piezo2 comes from a different pathway according to a recent theory based on reanalysis of an ALS genetic database [55,57].

Correspondingly, it was recently postulated that the channelopathy of Piezo2 could impair the Piezo system or, more precisely, the delicate crosstalk between Piezo1 and Piezo2 channels, which could lead to impaired orthostasis and proprioception [24,48]. Furthermore, the aforementioned theoretical entrainment mechanism could also be impaired due to this underlying Piezo system impairment and resultant neuro-energetic reallocation.

## 5. Conclusions

HRV is a widely accepted measure for monitoring the autonomic nervous system. HRV measurement has gained significant demand not only in science, but also in the public, due to the fairly low price and wide accessibility of the Internet of things. Therefore, the scientifically well-substantiated interpretation of unknown measures of HRV, such as LF power, is highly desired. The current author suggests that the earlier-observed undetectable value of LF power at medium- to high-intensity exercise reflects the inactivation of Piezo2 in the baroreceptors. Consequently, the current manuscript also proposes that the low-frequency power of the heart rate variability could represent the activity level of Piezo2 in baroreceptors. This interpretation could not only facilitate scientific research in order to establish this theory, but also help clinicians, rehabilitation experts, athletes, coaches, and the general public to use it as a threshold measure in certain instances. LF monitoring could gain importance in these special instances, e.g., in eccentric training in chronic heart failure, because it was recently proposed that Piezo2 in proprioceptors could go through pathological hyperexcitation, resulting in autonomic imbalance.

This opinion piece is not meant to challenge any earlier scientific finding, but rather incorporate them by also applying published Piezo-related theories with a neurocentric and interdisciplinary approach. Furthermore, ion channels other than Piezo and non-neural factors could certainly contribute to LF power in a secondary fashion, but this opinion piece focused on the suggested principal mechanism involving the Piezo2 ion channel, since the optogenetically eliminated Piezo2-containing neurons are those that could terminate the activity of baroreceptors. In addition, the features of Piezo2, such as their excitatory nature, abrupt activation, or potential inactivation in a hyperexcited state, as well as the fact that Piezo2-containing neurons may have intrinsic resonance properties, not to mention their presumed crosstalk with Piezo1, all point to the possibility that their activity level could reflect the LF power of HRV. Emerging optogenetic manipulation techniques in combination with power spectral analysis of HRV could reveal the proposed mechanism in play in the future.

## Data Availability

Not applicable.
